# Direct Detection and Differentiation of Pathogenic *Leptospira* Species Using a Multi-Gene Targeted Real Time PCR Approach

**DOI:** 10.1371/journal.pone.0112312

**Published:** 2014-11-14

**Authors:** Ana Sofia Ferreira, Pedro Costa, Teresa Rocha, Ana Amaro, Maria Luísa Vieira, Ahmed Ahmed, Gertrude Thompson, Rudy A. Hartskeerl, João Inácio

**Affiliations:** 1 Instituto Nacional de Investigação Agrária e Veterinária, I.P. (INIAV, I.P.), Unidade Estratégica de Investigação e Serviços em Produção e Saúde Animal, Lisboa, Portugal; 2 Instituto de Ciências Biomédicas de Abel Salazar, Universidade do Porto, Porto, Portugal; 3 Unidade de Microbiologia Médica, Instituto de Higiene e Medicina Tropical, Universidade Nova de Lisboa, Lisboa, Portugal; 4 Research Center in Biodiversity and Genetic Resources (CIBIO-ICETA), Universidade do Porto, Porto, Portugal; 5 WHO/FAO/OIE and National Collaborating Centre for Reference and Research on Leptospirosis, KIT Biomedical Research, Amsterdam, The Netherlands; 6 School of Pharmacy and Biomolecular Sciences, University of Brighton, Brighton, United Kingdom; University of Kentucky College of Medicine, United States of America

## Abstract

Leptospirosis is a growing public and veterinary health concern caused by pathogenic species of *Leptospira*. Rapid and reliable laboratory tests for the direct detection of leptospiral infections in animals are in high demand not only to improve diagnosis but also for understanding the epidemiology of the disease. In this work we describe a novel and simple *TaqMan*-based multi-gene targeted real-time PCR approach able to detect and differentiate *Leptospira interrogans*, *L. kirschneri*, *L. borgpeteresenii* and *L. noguchii*, which constitute the veterinary most relevant pathogenic species of *Leptospira*. The method uses sets of species-specific probes, and respective flanking primers, designed from *ompL1* and s*ecY* gene sequences. To monitor the presence of inhibitors, a duplex amplification assay targeting both the mammal *β-actin* and the leptospiral *lipL32* genes was implemented. The analytical sensitivity of all primer and probe sets was estimated to be <10 genome equivalents (GE) in the reaction mixture. Application of the amplification reactions on genomic DNA from a variety of pathogenic and non-pathogenic *Leptospira* strains and other non-related bacteria revealed a 100% analytical specificity. Additionally, pathogenic leptospires were successfully detected in five out of 29 tissue samples from animals (*Mus* spp., *Rattus* spp., *Dolichotis patagonum* and *Sus domesticus*). Two samples were infected with *L. borgpetersenii*, two with *L. interrogans* and one with *L. kirschneri*. The possibility to detect and identify these pathogenic agents to the species level in domestic and wildlife animals reinforces the diagnostic information and will enhance our understanding of the epidemiology of leptopirosis.

## Introduction

Leptospirosis is a growing and underestimated public health and veterinary concern, caused by pathogenic spirochetes belonging to the family *Leptospiracea*, genus *Leptospira* [1], [2]. The disease is an important cause of abortion, stillbirths, infertility, poor milk production and death amongst livestock, harboring a significant economic impact [3]–[5]. Its transmission requires circulation of the agents among domestic and wild animal reservoirs, with rodents recognized as the most important sources that establish persistent renal carriage and urinary shedding of *Leptospira*. Humans are incidental hosts acquiring a systemic infection upon direct or indirect exposure to the urine, blood or tissue of an infected animal. Farmers, veterinarians, sewer workers, pet keepers, rodent catchers and those persons participating in aquatic leisure activities are more prone to acquire the disease.

Conventional classification of *Leptospira* is based on serological criteria, using the serovar as the basic taxon. To date over 250 pathogenic serovars separated into 25 serogroups are known [6]. The serological classification system is complemented by a genotypic one, in which 21 genetic species are currently recognized, including pathogenic, intermediate and non-pathogenic (or saprophytic) species [7]–[10]. Genetic species boundaries hardly correlate with the serological classification [8].

Serological approaches are used commonly for diagnosis of leptospirosis in animals. The reference method is the Microscopic Agglutination Test (MAT), which has the advantage of being specific for serogroups [3] but has several drawbacks of being laborious and requiring a panel of viable *Leptospira* cultures. Isolation of leptospires, from suspect clinical specimens, constitutes the definitive diagnosis but is also technically demanding, time consuming and subject to contamination and high rates of failure [4]. Isolates are traditionally classified to the serovar level by the Cross Agglutinin Absorption Test (CAAT) [8] which is cumbersome for routine use and is only performed in a few reference laboratories worldwide.

Rapid and reliable laboratory tests for the direct detection of leptospiral infections in animals are in high demand, particularly to support suitable control measures. Serology does not corroborate well with the presence of pathogenic viable leptospires in the kidneys or urine and detection of the agents is necessary to identify healthy animal carriers. Molecular-based assays have been previously described for detecting leptospires in clinical samples. Most approaches are PCR-based and target specific genes or polymorphisms in the genome of pathogenic leptospires. Several real time PCR assays have been described predominantly for use with human samples such as whole-blood, serum or urine [11]–[17] but only few have been plentifully validated [18],[19]. A few assays were evaluated or used for detecting *Leptospira* in kidney tissue, blood, urine and other clinical specimens from animals such as sheep [20], dogs [21], [22], pigs [5], deer [23], flying foxes [24] and rodents [25], [26]. Most assays rely on SYBR green detection chemistry and only differentiate between pathogenic and non-pathogenic leptospires, lacking the ability to distinguish between different species. Nevertheless, speciation of infecting *Leptospira* from clinical material may be important for determining the clinical significance, the probable source of infection, to distinguish sporadic cases from possible outbreaks and to better access the epidemiology of the disease.

In the present work we have developed a novel and simple *TaqMan*-based multi-gene targeted real-time PCR approach yielding high sensitivity and specificity for the direct detection and differentiation of the most relevant pathogenic *Leptospira* species in animal samples, suitable for introduction into the routine diagnostics of veterinary laboratories.

## Materials and Methods

### Bacterial strains

Eighty five reference strains and clinical and environmental isolates of *Leptospira* spp. belonging to pathogenic, intermediate and non-pathogenic phylogenetic clades were used in this study (Table 1). Strains were obtained from the collection maintained by the *Instituto Nacional de Investigação Agrária e Veterinária* (INIAV), Portugal, which is the Portuguese reference laboratory for animal diseases, from the Leptospirosis Laboratory at the *Instituto de Higiene e Medicina Tropical* (IHMT/UNL), Portugal, and from the WHO/FAO/OIE and National Leptospirosis Reference Centre in Amsterdam, The Netherlands. Strains were grown in liquid Ellinghausen-McCullough-Johnson-Harris (EMJH) medium for up to 7 days.

**Table 1:** *Leptospira* strains used in the present study and results of the real time PCR assays using the species-specific probes and flanking primers.

Species	Serogroup	Serovar	Strain	Source^1^	Set 1^2^	Set 2^3^	Set 3^4^	Set 4^5^	Set 5^6^
*L. interrogans*	Australis	Muenchen	München C 90	KIT	+	+	-	-	-
	Australis	Australis	Ballico	KIT	+	+	-	-	-
	Australis	Bratislava	Jez Bratislava	INIAV	+	+	-	-	-
	Autumnalis	Autumnalis	Akiyami A	INIAV	+	+	-	-	-
	Bataviae	Bataviae	Van Tienem	INIAV	+	+	-	-	-
	Canicola	Canicola	Hond Utrecht IV	INIAV	+	+	-	-	-
	Djasiman	Djasiman	Djasiman	KIT	+	+	-	-	-
	Hebdomadis	Hebdomadis	Hebdomadis	KIT	+	+	-	-	-
	Hebdomadis	Kremastos	Kremastos	KIT	+	+	-	-	-
	Icterohaemorrhagiae	Birkini	Birkin	KIT	+	+	-	-	-
	Icterohaemorrhagiae	Copenhageni	M20	INIAV	+	+	-	-	-
	Icterohaemorrhagiae	Icterohaemorrhagiae	RGA	INIAV	+	+	-	-	-
	Icterohaemorrhagiae	Lai	Lai	KIT	+	+	-	-	-
	Pomona	Pomona	Pomona	INIAV	+	+	-	-	-
	Pyrogenes	Pyrogenes	Salinem	INIAV	+	+	-	-	-
	Sejroe	Hardjo type Prajitno	Hardjoprajitno	IHMT	+	+	-	-	-
*L. borgpetersenii*	Ballum	Ballum	Mus 127	INIAV	+	-	+	-	-
	Ballum	Castellonis	Castellon 3	KIT	+	-	+	-	-
	Hebdomadis	Jules	Jules	KIT	+	-	+	-	-
	Hebdomadis	Worsfoldi	Worsfold	KIT	+	-	+	-	-
	Javanica	Ceylonica	Piyasena	KIT	+	-	+	-	-
	Javanica	Poi	Poi	INIAV	+	-	+	-	-
	Javanica	Zhenkang	L 82	KIT	+	-	+	-	-
	Mini	Mini	Sari	IHMT	+	-	+	-	-
	Pyrogenes	Kwale	Julu	KIT	+	-	+	-	-
	Sejroe	Hardjo type bovis	Sponselee	KIT	+	-	+	-	-
	Sejroe	Hardjo type bovis	L550	KIT	+	-	+	-	-
	Sejroe	Hardjo type bovis	JB197	KIT	+	-	+	-	-
	Sejroe	Nyanza	Kibos	KIT	+	-	+	-	-
	Sejroe	Sejroe	M84	KIT	+	-	+	-	-
	Tarassovi	Kisuba	Kisuba	KIT	+	-	+	-	-
	Tarassovi	Tarassovi	Mitis Johnson	INIAV	+	-	+	-	-
*L. kirschneri*	Australis	Ramisi	Musa	KIT	+	-	-	+	-
	Autumnalis	Bulgarica	Nicolaevo	KIT	+	-	-	+	-
	Autumnalis	Butembo	Butembo	KIT	+	-	-	+	-
	Cynopteri	Cynopteri	3522C	IHMT	+	-	-	+	-
	Grippotyphosa	Grippotyphosa type Moskva	Moskva V	IHMT	+	-	-	+	-
	Grippotyphosa	Ratnapura	Wumalasena	KIT	+	-	-	+	-
	Grippotyphosa	Vanderhoedeni	Kipod 179	KIT	+	-	-	+	-
	Icterohaemorrhagiae	Bogvere	LT 60-69	KIT	+	-	-	+	-
	Pomona	Mozdok	5621	KIT	+	-	-	+	-
	Pomona	Mozdok	Portugal 1990	INIAV	+	-	-	+	-
	Pomona	Tsaratsovo	B 81/7	KIT	+	-	-	+	-
*L. noguchii*	Australis	Nicaragua	1011	KIT	+	-	-	-	+
	Autumnalis	Fortbragg	Fort Bragg	KIT	+	-	-	-	+
	Bataviae	Argentiniensis	Peludo	KIT	+	-	-	-	+
	Djasiman	Huallaga	M 7	KIT	+	-	-	-	+
	Louisiana	Louisiana	LSU 1945	KIT	+	-	-	-	+
	Panama	Panama	CZ 214	INIAV	+	-	-	-	+
	Pomona	Proechimys	1161 U	KIT	+	-	-	-	+
	Pyrogenes	Myocastoris	LSU 1551	KIT	+	-	-	-	+
	Shermani	Carimagua	9160	KIT	+	-	-	-	+
*L. santarosai*	Ballum	Peru	MW 10	KIT	+	-	-	-	-
	Bataviae	Balboa	735 U	KIT	+	-	-	-	-
	Bataviae	Kobbe	CZ 320	KIT	+	-	-	-	-
	Grippotyphosa	Canalzonae	CZ 188	KIT	+	-	-	-	-
	Hebdomadis	Borincana	HS 622	KIT	+	-	-	-	-
	Hebdomadis	Maru	CZ 285	KIT	+	-	-	-	-
	Javanica	Fluminense	Aa 3	KIT	+	-	-	-	-
	Mini	Beye	1537 U	KIT	+	-	-	-	-
	Sarmin	Rio	Rr 5	KIT	+	-	-	-	-
	Sejroe	Guaricura	Bov.G.	KIT	+	-	-	-	-
	Shermani	Babudieri	CI 40	KIT	+	-	-	-	-
	Shermani	Shermani	1342 K	KIT	+	-	-	-	-
	Tarassovi	Atchafalaya	LSU 1013	KIT	+	-	-	-	-
*L. weilii*	Celledoni	Celledoni	Celledoni	INIAV	+	-	-	-	-
	Celledoni	Mengding	M 6906	KIT	+	-	-	-	-
	Javanica	Coxi	Cox	KIT	+	-	-	-	-
	Javanica	Mengma	S 590	KIT	+	-	-	-	-
	Javanica	Mengrun	A 102	KIT	+	-	-	-	-
	Mini	Hekou	H 27	KIT	+	-	-	-	-
	Pyrogenes	Menglian	S 621	KIT	+	-	-	-	-
	Sarmin	Sarmin	Sarmin	KIT	+	-	-	-	-
	Tarassovi	Topaz	94-79970/3	KIT	+	-	-	-	-
	Tarassovi	Vughia	LT 89-68	KIT	+	-	-	-	-
*L. alexanderi*	Hebdomadis	Manzhuang	A 23	KIT	nd	-	-	-	-
	Javanica	Mengla	A 85	KIT	nd	-	-	-	-
	Manhao	Manhao 3	L 60	KIT	nd	-	-	-	-
	Mini	Yunnan	A 10	KIT	nd	-	-	-	-
*L. meyeri*	Ranarum	Ranarum	ICF	KIT	nd	-	-	-	-
	Semaranga	Semaranga	Veldrat Semaranga 173	KIT	nd	-	-	-	-
*L. inadai*	Manhao	Lincang	L 14	KIT	nd	-	-	-	-
*L.fainei*	Hurstbridge	Hurstbridge	BUT 6T	KIT	nd	-	-	-	-
*L.biflexa*	Andaman	Andamana	CH 11	KIT	-	-	-	-	-
	Semaranga	Patoc	Patoc I	KIT	-	-	-	-	-

1 INIAV - Instituto Nacional de Investigação Agrária e Veterinária, Lisbon, Portugal. IHMT - Instituto de Higiene e Medicina Tropical, Lisbon, Portugal. KIT - Royal Tropical Institute, Amsterdam, The Netherlands;

2 Set 1 targets the *lipL32* gene of pathogenic *Leptospira* spp.;

3 Set 2 targets the *secY* gene of *L. interrogans*;

4 Set 3 targets the *ompL1* gene of *L. borgpetersenii*;

5 Set 4 targets the *secY* gene of *L. kirschneri*;

6 Set 5 targets the *secY* gene of *L. noguchii*; nd - not done; Amplification (+) or no amplification (−).

Culturing *Leptospira* from tissue samples was performed as described by the OIE Manual of Diagnostic Tests and Vaccines for Terrestrial Animals [27]. Other bacterial strains were provided by INIAV for assessing the analytical specificity of the amplification reactions, representing the species: *Acinetobacter baumannii* (LNIV 1628/12), *Bacillus licheniformis* (VLA 1831), *Klebsiella pneumoniae* (VLA 1643), *Salmonella* Dublin (VLA 1272), *Streptococcus agalactiae* (VLA 33), *Proteus mirabilis* (LNIV 2269/II), *Yersinia enterocolitica* (VLA 1884), *Staphylococcus aureus* (VLA 1032), *Pseudomonas aeruginosa* (VLA 67), *Arcanobacterium pyogenes* (VLA 1321) and *Listeria monocytogenes* (VLA 1774).

### Spiked tissue samples

A sample of kidney tissue from a bovine was used for testing as spiked sample. The kidney was acquired from a local official slaughterhouse (Raporal, Portugal), obtained from a bovine intended for normal human consumption, with no signs of leptospirosis. The bovine was not killed specifically for the purpose of this study. Approximately 200 mg portions of kidney tissue were excised with a sterile scalpel and homogenized with 5 ml of PBS buffer in a sterile plastic bag (Whirl-Pak bags) using a stomacher lab-blender. Kidney samples were individually spiked with the following strains, in order to determine the analytical detection sensitivity: *Leptospira interrogans* (serovar Autumnalis, strain Akiyami), *L. kirschneri* (serovar Mozdok, strain Portugal 1990) [28], *L. noguchii* (serovar Panama, strain CZ 214K) and *L. borgpetersenii* (serovar Tarassovi, strain Mitis Johnson). All the strains were grown at 29°C and the concentrations of leptospires were determined using a Petroff-Hausser counting chamber and adjusted to 10^8^ cells/ml with PBS buffer. For each strain, tenfold serial dilutions from 10^7^ to 10^0^ cells/ml were prepared in PBS buffer and 0.1 ml aliquots were used to spike 0.9 ml of tissue homogenates. Tissue homogenate spiked with 0.1 ml PBS buffer was used as negative control. DNA extraction was performed as described in the paragraph “Genomic DNA extraction” below.

### Tissue samples

INIAV IP is the Portuguese Reference Laboratory for animal diseases and provides diagnostic services to national veterinary authorities and private clients. Twenty seven dead wild rodents (25 *Mus* spp. and 2 *Rattus* spp.) were sent to the INIAV laboratory during the year 2011 for analysis and further used in this study (Table 2). The rodents were captured in the Lisbon Zoo under routine operations for rodent population control, by the local veterinary authorities. No animals were sacrificed for the only purposes of research. Additionally, a Patagonian mara (*Dolichotis patagonum*), also from the zoo, and a swine (*Sus domesticus*) stillbirth fetus, from a private client, both suspect of dying with leptospirosis, were submitted for analysis to our reference laboratory and later included in this study (Table 2). On arrival to the laboratory, animals were given a reference number and sent to the pathology where kidney, liver and/or lung tissue samples were collected. Specimens were then analysed using culture-based methods according to the OIE standard procedures for leptospirosis [27]. Briefly, specimens were aseptically collected at necropsy, immediately emulsified in sterile buffered saline solution in a 10% tissue suspension, two to three drops were inoculated in a first tube of medium and two more tubes were similarly inoculated with increasing 10-fold dilutions of the tissue suspension. For the tissue culture, a semisolid *Leptospira* EMJH medium was used by adding 0.1% agar to commercial EMJH (Difco), to which rabbit serum (0.4%) and 5-Fluorouracil (100 µg/ml) were further added [27].

**Table 2:** Results of the bacteriological culture and of the real time amplification assays for the tissue samples analyzed in the present study.

Sample	Origin	Set Actin^1^	Set 1^2^	Set 2^3^	Set 3^4^	Set 4^5^	Set 5^6^	Bacteriological analysis^7^
12-17433-Z1	*Mus* sp.	+	+	-	+	-	-	*L. borgpetersenii*
12-18078-Z6	*Mus* sp.	+	+	-	+	-	-	*L. borgpetersenii*
12-18458-Z13	*Mus* sp.	+	-	-	-	-	-	Negative
12-18458-Z14	*Mus* sp.	+	-	-	-	-	-	Negative
12-19472-Z15	*Mus* sp.	+	-	-	-	-	-	Negative
12-20553-Z16	*Mus* sp.	+	-	-	-	-	-	Negative
12-22955-Z17	*Mus* sp.	+	-	-	-	-	-	Negative
12-22955-Z18	*Mus* sp.	+	-	-	-	-	-	Negative
12-22955-Z19	*Mus* sp.	+	-	-	-	-	-	Negative
12-22955-Z20	*Mus* sp.	+	-	-	-	-	-	Negative
12-22955-Z22	*Mus* sp.	+	-	-	-	-	-	Negative
12-22955-Z23	*Mus* sp.	+	-	-	-	-	-	Negative
12-22955-Z24	*Mus* sp.	+	-	-	-	-	-	Negative
12-22955-Z25	*Mus* sp.	+	-	-	-	-	-	Negative
12-22955-Z26	*Mus* sp.	+	-	-	-	-	-	Negative
12-22955-Z27	*Mus* sp.	+	-	-	-	-	-	Negative
12-22955-Z28	*Mus* sp.	+	-	-	-	-	-	Negative
12-22955-Z29	*Mus* sp.	+	-	-	-	-	-	Negative
12-22955-Z30	*Mus* sp.	+	-	-	-	-	-	Negative
12-22955-Z31	*Mus* sp.	+	-	-	-	-	-	Negative
12-22955-Z32	*Mus* sp.	+	-	-	-	-	-	Negative
12-22955-Z33	*Mus* sp.	+	-	-	-	-	-	Negative
12-22955-Z34	*Mus* sp.	+	-	-	-	-	-	Negative
12-22955-Z36	*Mus* sp.	+	-	-	-	-	-	Negative
12-22955-Z37	*Rattus* sp.	+	+	+	-	-	-	*L. interrogans*
12-22955-Z38	*Mus* sp.	+	-	-	-	-	-	Negative
12-22955-Z39	*Rattus* sp.	+	-	-	-	-	-	Negative
11-36840	*Dolichotis patagonum*	+	+	+	-	-	-	*L. interrogans*
12-494	*Sus domesticus* (fetus)	+	+	-	-	+	-	*L. kirschneri*

1 Set Actin targets the *β-actin* gene of mammals,

2 Set 1 targets the *lipL32* gene of pathogenic *Leptospira*;

3 Set 2 targets the *secY* gene of *L. interrogans*;

4 Set 3 targets the *ompL1* gene of *L. borgpetersenii*;

5 Set 4 targets the *secY* gene of *L. kirschneri*;

6 Set 5 targets the *secY* gene of *L. noguchii*;

7 The analysis of the partial sequences of the *secY* gene of each isolate allowed to identify the *Leptospira* species; Amplification (+) or no amplification (−).

DNA was extracted directly from tissues homogenates as described below.

### Genomic DNA extraction

Genomic DNA was extracted from both bacterial liquid cultures and tissue homogenates using the QIAamp DNA extraction kit according to the manufacturer's instructions (Qiagen, Hilden, Germany), with a final elution volume of 200 µl. The DNA concentration from the pure cultures was estimated spectrophotometrically using a Nanodrop 1000 spectrophotometer (Nanodrop Technologies, Wilmington, DE) and standardized to a concentration of 10^4^ genome equivalents (GE)/µl for use in the reactions. The number of GE was estimated using an average genome size of 4.6 Mb [29]. Genomic DNA suspensions were stored at −20°C until further use.

### Design of *TaqMan* probes and flanking primers

DNA sequences of representative strains and species of *Leptospira* were retrieved from NCBI-GenBank and aligned using the ClustalW algorithm implemented in the program MegAlign (vers. 5.03) (DNAStar, USA). Primers and dual labeled hydrolysis probes (*TaqMan* probes) were designed to target selected species-specific genetic polymorphisms of the following pathogenic *Leptospira* spp.: *L. interrogans*, *L. borgpetersenii*, *L. kirschneri* and *L. noguchii* (Table 3). Probes and primers specificities were assessed *in silico* using the BLAST tools from NCBI-GenBank. All probes and primers were synthesized by MWG Biotech (Germany).

**Table 3:** Primers and probes used in this study targeting selected genes of pathogenic species of *Leptospira*.

Set	Primer/Probe	Sequence (5′- 3′)	Annealing temperature	Complementary target species
**Set**	F_Actin	GGC TCY ATY CTG GCC TC	60°C	*β-actin* gene of mammals
**Actin** 1	R_Actin	GCA YTT GCG GTG SAC RAT G		
	P_Actin	Cy5.5 (Quasar 705) -TAC TCC TGC TTG CTG ATC CAC ATC-BHQ2		
**Set 1** 2	45F	AAG CAT TAC CGC TTG TGG TG	60°C	*lipL32* gene of pathogenic *Leptospira* spp.
	286R	GAA CTC CCA TTT CAG CGA TT		
	taq-189P	FAM-AAA GCC AGG ACA AGC GCC G-BHQ1		
**Set 2**	PFLint2	CTT GAG CCT GCG CGT TAY C	63°C	*secY* gene of *L. interrogans*
	PRLint2	CCG ATA ATT CCA GCG AAG ATC		
	TaqLint2	TET-CTC ATT TGG TTA GGA GAA CAG ATC A-BHQ1		
**Set 3**	F_bpn	GAT TCG GGT TAC AAT TAG ACC	65°C	*ompL1* gene of *L. borgpetersenii*
	R_bpn1	TTG ATC TAA CCG GAC CAT AGT		
	TqM_bpn	Cy5.5 (Quasar 705) -TAC TAA GGA TGG TTT GGA CGC TGC-BHQ2		
**Set 4**	F_nery	CTG GCT TAA TCA ATG CTT CTG	60°C	*secY* gene of *L. kirschneri*
	R_nery	CTC TTT CGG TGA TCT GTT CC		
	TqM_nery	Texas Red-CAG TTC CAG TTG TAA TAG ATA AGA TTC-BHQ2		
**Set 5**	FLnog2	TCA GGG TGT AAG AAA GGT TC	63°C	*secY* gene of *L. noguchii*
	RLnog2	CAA AAT TAA AGA AGA AGC AAA GAT		
	TaqLnog	FAM-CGA TTG GCT TTT TGC TTG AAC CATC-BHQ1		

1 Retrieved from Costa et al. [31];

2 Retrieved from Stoddard et al. [16].

### Real-time PCR assays

We have implemented the following assay format for testing DNA templates extracted from biological samples: (i) a first duplex amplification step aiming the detection of pathogenic *Leptospira* spp. (by targeting the leptospiral *lipL32* gene; Table 3) and including an internal control to monitor the presence of potential amplification inhibitors (by targeting the mammal *β-actin* gene; Table 3); (ii) if pathogenic leptospires are detected in the first reaction, these may be further discriminated by testing each of the *L. interrogans*, *L. borgpetersenii*, *L. kirschneri* and *L. noguchii* targeted probes/primers (Table 3). The CFX96 real-time PCR detection system (Bio-Rad, USA) was used for all assays. The amplification reactions were optimized individually for all the probes and associated primers using the SsoFast Probes Supermix (Bio-Rad, USA), according to the manufacturer's instructions. Each reaction was conducted in a total volume of 20 µl consisting of 1× SsoFast Probes Supermix, 400 nM of each primer, 150 nM of *TaqMan* probe, DNase free water (GIBCO) and 5 µl of DNA template solution (extracted from pure cultures or tissues samples). Non-template negative controls (with PCR grade water) were included in each run to rule out the possibility of cross-contamination. The assay thermal conditions were as follows: 95°C for 2 min, followed by 45 cycles of 5 s at 95°C and 15 s at the optimized annealing temperature for each probe (Table 3). The thermal cycling conditions for the duplex amplification targeting *β-actin* and *lipL32* were 95°C for 2 min, followed by 45 cycles of 5 s at 95°C and 35 s at 60°C. Reproducibility of the assays was assessed by repeating the assays at least twice. Data analyses were performed by the detection system of the real-time PCR equipment, according to the manufacturer's instructions.

### Analytical specificity and sensitivity

In order to determine if each set of probe and associated primers was specific for the respective *Leptospira* target species, the amplification assays were tested on DNA templates extracted from different strains belonging to pathogenic, intermediate and non-pathogenic *Leptospira* species (Table 1), and from other non-related bacteria previously mentioned in “bacterial strains” section. The analytical sensitivity of the amplification assays (limits of detection – LODs) were determined using 10-fold serial dilutions of genomic DNA extracted from pure cultures of *L. interrogans* (serovar Autumnalis, strain Akiyami), *L. kirschneri* (serovar Mozdok, strain Portugal 1990), *L. noguchii* (serovar Panama, strain CZ 214K) and *L. borgpetersenii* (serovar Tarassovi, strain Mitis Johnson). LODs on tissue samples were assessed using DNA extracted from the serially diluted spiked macerates. Each template was tested in triplicate.

### Sequencing

*Leptospira* isolates obtained from tissue samples were identified by comparative sequence analysis of a 245 bp region of the s*ecY* gene, as described by Victoria *et al.* [30]. Briefly, the region of interest was amplified using primers SecYII (5′-GAA TTT CTC TTT TGA TCT TCG-3′) and SecYIV (5′-GAG TTA GAG CTC AAA TCT AAG-3′), which amplify *secY* sequences from all pathogenic strains of *Leptospira*. PCR amplifications were performed on a C1000 thermocycler (Bio-Rad) using the following program: an initial step of denaturation for 5 min at 95°C, followed by 34 cycles consisting of annealing, 45 sec at 54°C, extension, 2 min at 72°C, and denaturation, 30 sec at 94°C. Nucleotide sequences were determined, using the same primers, by commercially available sequencing services. Nucleotide sequence analysis and comparison with other relevant reference sequences were performed using the BLAST suite at NCBI-GenBank and aligned using Clustal X or MEGA software (version 5.0).

## Results

### Design of probes and primers

Species-specific sets of primers and probes targeting *L. interrogans*, *L. borgpetersenii*, *L. kirschneri* and *L. noguchii* are listed in Table 3. As shown in Figures S1, S2, S3 and S4 in File S1, these sets of probes and primers contained sufficient polymorphisms to warrant ‘*in silico*’ species specific amplification.

### Analytical specificity and sensitivity

**Figure 1:**
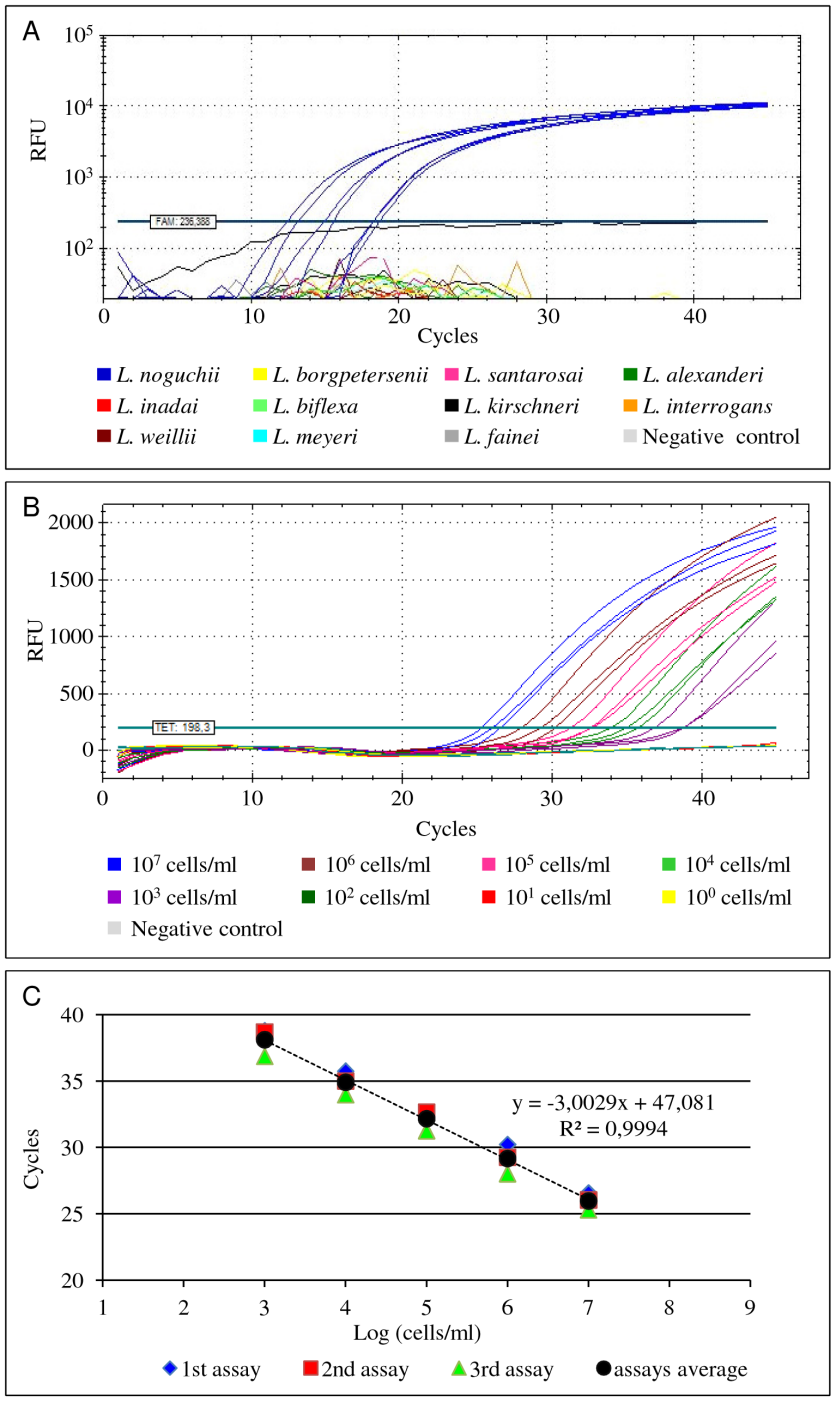
Illustration of the real-time PCR amplification curves obtained during the optimization of the assays. (A) Specificity tests of the *L. noguchii* targeted amplification assay using the TaqLnog probe combined with the flanking primers FLnog2 and RLnog2. Blue amplification curves represent *L. noguchii* strains. All other non-target strains yielded no amplification results. (B) Estimation of the limit of detection of the amplification assay targeting *L. interrogans* (serovar Autumnalis, strain Akiyami) using DNA extracted directly from spiked bovine kidney samples as template as a typical example of all *Leptospira* probe and primer sets. The amplification curves obtained from different ten-fold serial dilutions of the target *Leptospira* are represented by different colours. Unspiked tissue homogenate (grey line) was used as negative control. (C) Standard curve obtained from the analysis of the amplification curves mentioned in the previous panel B. RFU - Relative Fluorescence Units.

Execution of the PCRs on DNA extracted from various bacteria, revealed a highly specific amplification from any of the pathogenic strains belonging to the respective target *Leptospira* spp., i.e. *L. interrogans*, *L. kirschneri*, *L borgpetersenii* and *L. noguchii*. None of the other strains yielded a positive amplification reaction (Table 1; Fig. 1A). The analytical sensitivity (LOD) of the amplification assays were found to be between 1 and 10 genome copies in the PCR mixture for each probe and primer set.

### Spiked tissue samples

The LOD of the PCRs on spiked tissue samples was similar for all probe/primers sets targeting the respective target species, and estimated to be 10^3^ leptospires/ml of tissue homogenate (≈ per 20 mg of tissue) (Fig. 1B). Furthermore, the same LOD was estimated for the *lipL32*-targeted probe/primers when used in duplex amplification reactions with the mammal *β-actin* probe (not shown).

### Clinical tissue samples

**Figure 2:**
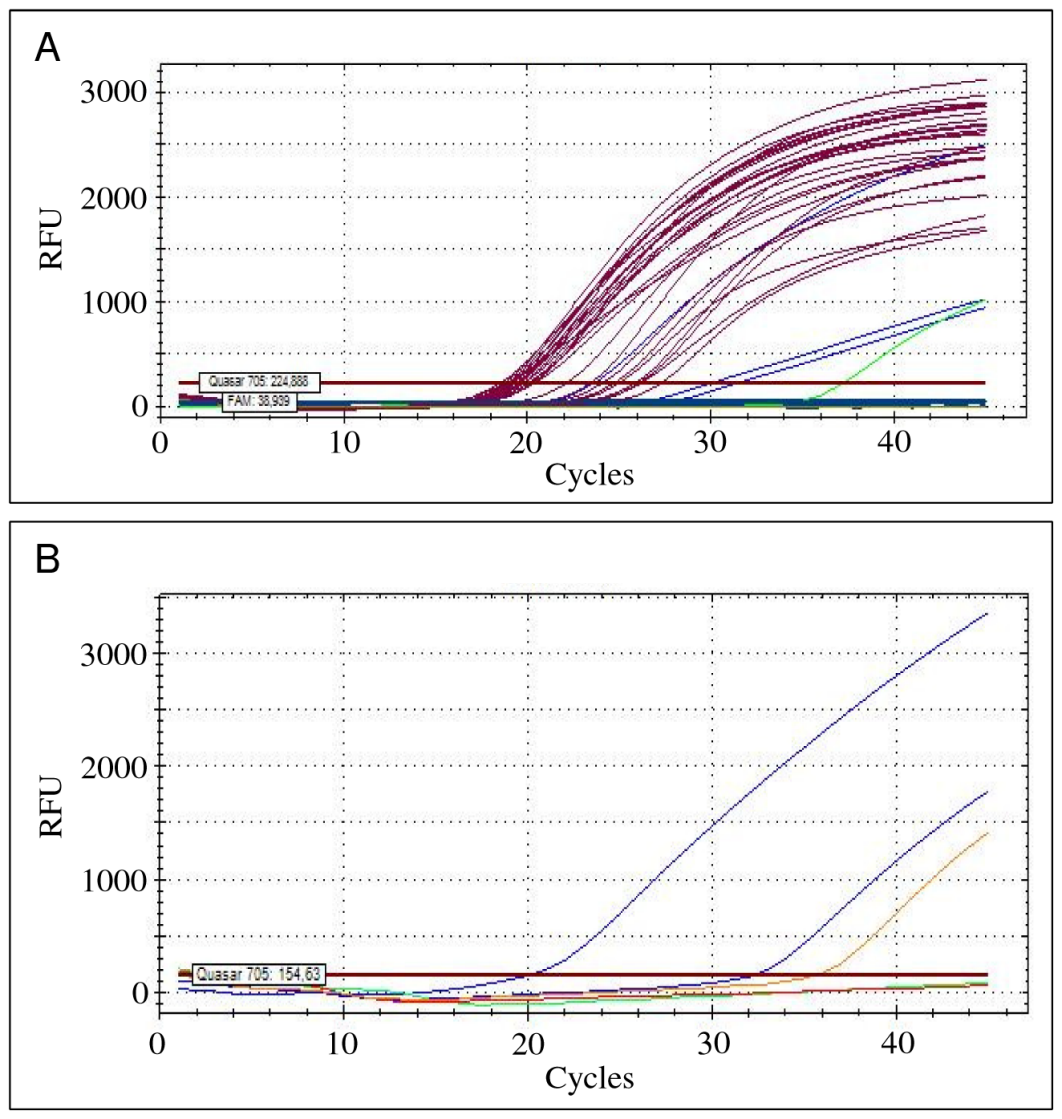
Illustration of the real-time PCR amplification curves obtained during the testing of naturally-infected tissue samples. (A) Results of the *β-actin* and *lipL32* targeted duplex amplification assay when testing representative samples from the wild rodents. The partial *β-actin* gene was amplified from all tissue samples (dark pink lines). Leptospiral DNA was detected in three samples by a positive amplification of the *lipL32* gene (blue lines). A spiked positive control with *L. interrogans* (serovar Autumnalis, strain Akiyami) is shown (green line). (B) From the previous leptospiral positive amplification results, two samples were assessed as infected with *L. borgpetersenii* using the respective targeted amplification assay with probe TqM_bpn and flanking primers F_bpn and R_bpn1 (blue lines). The positive and negative controls are illustrated by the orange and red lines, respectively.

DNA extracted from 27 kidney samples of wild rodents were analysed with the *lipL32* and mammal *β-actin* targeted duplex assay (Table 2; Fig. 2A). Leptospiral DNA was detected in three samples, as demonstrated by a positive amplification of the *lipL32* gene region (Table 2; Fig. 2A). Furthermore, the partial *β-actin* gene was amplified from all samples, showing that the PCR reactions were not significantly inhibited by potential contaminants. When tested with each of the *L. interrogans*, *L. borgpetersenii*, *L. kirschneri* and *L. noguchii* targeted probes/primers, only these three samples showed amplification (Table 2; Fig. 2B). Two of these DNA samples were identified as *L. borgpetersenii* and one sample as *L. interrogans*. Testing a pooled sample of kidney and liver tissues from a Patagonian mara, and a lung sample from an aborted swine fetus with the duplex PCR revealed a positive amplification for both samples (Table 2). Subsequent testing with the species-specific sets of probes and primers showed that the Patagonian mara was infected with *L. interrogans* and the swine fetus with *L. kirschneri*.

*Leptospira* isolates were only cultured from the samples that also yielded PCR-positive results, thus confirming the presence of viable leptospires (Table 2).

Molecular speciation through analysis of the partial sequences of the *secY* gene was in concordance with the results obtained by the species-specific PCRs. Two isolates were identified as *L. borgpetersenii* (from wild rodents; GenBank accession numbers KM066006 and KM066007), one as *L. kirschneri* (from the swine fetus; accession number KM066009) and two as *L. interrogans* (from a wild rodent and the Patagonian mara; accession numbers KM066008 and KM066010, respectively).

## Discussion

In this work we present a two step real-time PCR strategy to infer the presence of pathogenic leptospires in clinical and veterinary samples. In the first step, we assess if an animal tissue sample is infected with a pathogenic leptospire by targeting its *lipL32* gene. The *lipL32* gene encodes an outer membrane lipoprotein that is confined to pathogenic *Leptospira* species [16]. The second step identifies the four most common and veterinary relevant pathogenic *Leptospira* species, *L. interrogans*, *L. borgpetersenii*, *L. kirschneri* and *L. noguchii* using dedicated sets of probes and primers.

Probes and flanking primers were developed by *in silico* analysis and further tested for their practical utility on DNA extracted from cultured bacteria, spiked tissues and clinical specimens. The amplification assays have proved to be specific to the respective targeted species, with no cross-reactions when non-pathogenic leptospires or other pathogens were tested. The amplification of the *β-actin* gene was included in the initial *lipL32*-based PCR to assess the presence of amplification inhibitors in tissue samples [31]. However, the abundant presence of *β-actin* gene copies in DNA samples extracted from tissues may ensure some amplification even when low levels of potential inhibitors are present (but amplification curves are usually weaker and anomalous). The analytical sensitivity deduced for the amplification assays, i.e. 1 to 10 GE on DNA extracted from cultured leptospires and 10^3^ leptospires/ml tissue homogenate, were similar to the ones of other previous studies concerning the molecular detection of leptospires [15]–[17], [19], [22].

The panel of species-specific probes and flanking primers may be extended with the design of novel oligonucleotides, e.g. for use in regions where the occurrence of additional species of pathogenic leptospires is common. As far as we know, this is the first report describing a strategy capable of clearly identify four most frequently found pathogenic *Leptospira* species based on the use of *TaqMan* probes.

From 27 kidney samples of wild rodents, and samples from a Patagonian mara and a porcine fetus suspected of leptospirosis, three rodent samples and the samples from the Patagonian mara and fetus all yielded a positive PCR test for the presence of pathogenic leptospires. In concordance, these samples were also positive by culture. Culture provides proof of infection and thus is an ideal reference standard. Consequently, these results are consistent with a 100% clinical sensitivity and specificity of the PCR. Subsequent prospective analysis of a larger sample set would allow substantiating this conclusion.

Phylogenetic identification of the cultures also allowed supporting the findings obtained with the species-specific PCRs. Indeed, speciation by phylogeny was in all cases in concordance with the results obtained via the PCR method.

Initially, we anticipated that more samples would be positive by the real time PCR assay than by culture [5], [32]–[34]. Recently, Fornazari *et al.* [20] reported that quantitative PCR presented the highest sensitivity among several techniques to detect leptospires in tissues samples, the bacteriological culture being the least sensitive. Apparently, our procedure of culturing, using macerated fresh tissue has been highly effective. Alternatively, it cannot be excluded that the bacterial load of the tissues might have been very high. Nevertheless, the low rate of positive animals (11%) is not too discrepant from the prevalence values found in other studies where leptospiral DNA was detected in rodents tissues by PCR-based assays, which ranged from 13% to 20% [25], [35], [36]. Furthermore, as far as we know, the region of Lisbon, where the rodents were captured, is not usually regarded as having major leptospirosis problems [2], which may also reflect a lower prevalence of the agent in reservoirs such as wild rodents. We anticipate that our assays may be useful in studies inferring the prevalence of pathogenic leptospires in wild rodents and other animals, with the advantage of differentiating the infecting *Leptospira* species.

The amplification assays described were able to detect pathogenic leptospires in samples of animal tissues, such as kidney or lung. Although the analysis of this kind of samples is not essential for an early diagnosis of leptospirosis, it has a great value in situations such as epidemiological and post-mortem investigations. The last situation is very well illustrated in this work with the detection of pathogenic leptospires in tissues of a Patagonian mara and a swine fetus. Both animals were suspect of having leptospirosis, which was confirmed by this study. The porcine fetus was infected with a strain belonging to *L. kirschneri*. Pigs may be infected by several *Leptospira* species (and serovars) that may cause infertility, fetal death and abortion. *Leptospira kirschneri* has been reported but seems to be less frequently found in pigs in Portugal than other species [37]. The Patagonian mara, a relatively large rodent that lived in the local zoo, was found to be infected with *L. interrogans*. To our knowledge, this is the first report describing the molecular detection or the isolation of a pathogenic leptospire from that rodent, which proved to have died of leptospirosis. Zoos are often infested with rats that are notorious reservoirs of *L. interrogans*. We hypothesise that this Patagonian mara has been infected by rats as the primary infection reservoir, which would support the potential hazard of rodents in zoos for both (exotics) animals and public.

The amplification assay described in this work is able to indentify the four most relevant pathogenic species of *Leptospira* infecting farm and wild animals. While the approach can be extended to other *Leptospira* species, it is important to continually evaluate the specificity of previously designed probes and primers and, if necessary, modify and improve the sequences, in order to ensure an effective and specific detection and identification of the circulating *Leptospira* species.

## Conclusions

The molecular assays presented in this work allow the detection and identification of four relevant pathogenic species of *Leptospira*, directly from animal tissues. The assays proved to be specific and sensitive, and much faster than the bacteriological culture, reducing the time for confirmatory leptospirosis diagnosis. The assays are amenable to future automation possibilities and will reinforce the diagnostic information and enhance our knowledge about the epidemiology of leptopirosis.

## Supporting Information

File S1 **Sequence alignments showing the complementary targets of the species-specific** ***Leptospira interrogans*** **,** ***L. kirschneri*** **,** ***L. noguchii*** **and** ***L. borgpetersenii*** **probes and respective flanking primers.**
(PDF)
