# Supplementary material for: Direct Detection and Differentiation of Pathogenic Leptospira Species Using a Multi-Gene Targeted Real Time PCR Approach
Source: PLoS One. 2014 Nov 14;9(11):e112312. doi: 10.1371/journal.pone.0112312 (PMC4232388; doi:10.1371/journal.pone.0112312)
Supplement: File S1 — Sequence alignments showing the complementary targets of the species-specific Leptospira interrogans , L. kirschneri , L. noguchii and L. borgpetersenii probes and respective flanking primers. (PDF) [file pone.0112312.s001.pdf]

Direct detection and differentiation of pathogenic *Leptospira* species using a multi-gene targeted real time PCR approach

Ana Sofia Ferreira[1,2], Pedro Costa[1,3], Teresa Rocha[1], Ana Amaro[1], Maria Luísa Vieira[3], Ahmed Ahmed[5], Gertrude Thompson[2,4], Rudy A. Hartskeerl[5], João Inácio[1,6]

[1]Instituto Nacional de Investigação Agrária e Veterinária, I.P. (INIAV, I.P.), Unidade Estratégica de Investigação e Serviços em Produção e Saúde Animal, Lisboa, Portugal; [2]Instituto de Ciências Biomédicas de Abel Salazar, Universidade do Porto, Porto, Portugal; [3]Unidade de Microbiologia Médica, Instituto de Higiene e Medicina Tropical, Universidade Nova de Lisboa, Lisboa, Portugal; [4]Research Center in Biodiversity and Genetic Resources (CIBIO-ICETA), Universidade do Porto, Portugal; [5]WHO/FAO/OIE and National Collaborating Centre for Reference and Research on Leptospirosis, KIT Biomedical Research, , Amsterdam, The Netherlands; [6]School of Pharmacy and Biomolecular Sciences, University of Brighton, Brighton, UK

Supporting Information

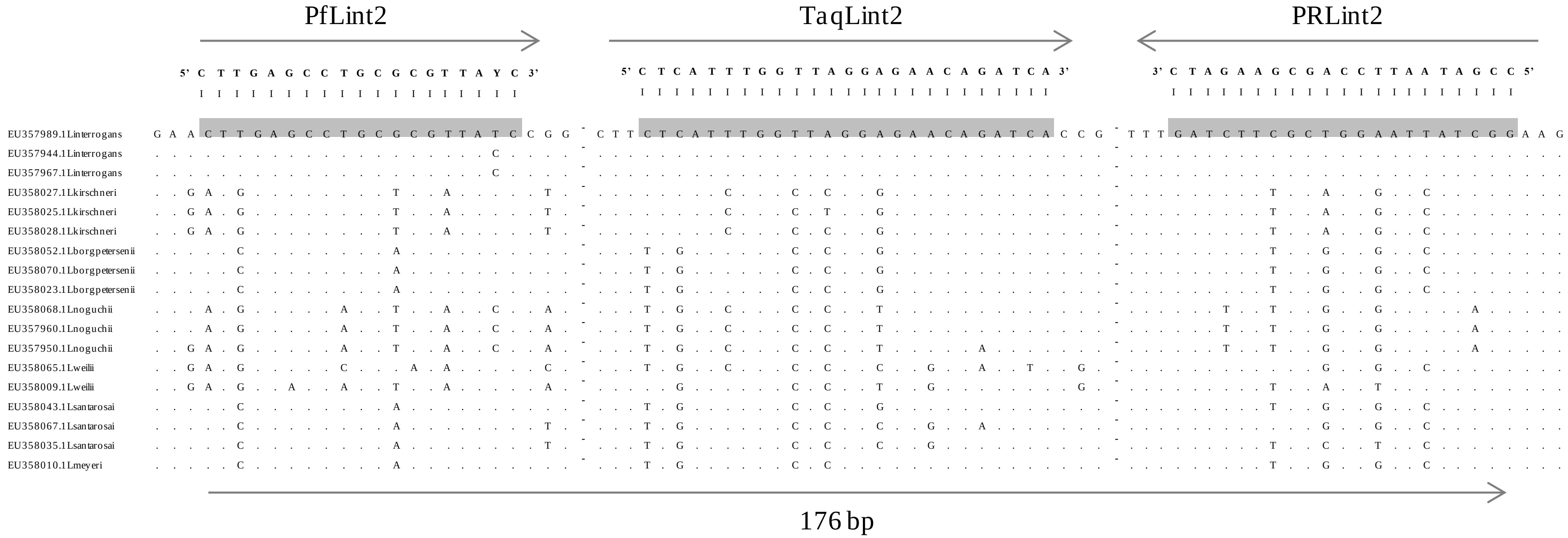

Figure S1. Complementary targets of the species-specific *Leptospira interrogans* probe and respective flanking primers. Partial alignment of *secY* gene sequences of representative *Leptospira* species showing the complementary targets of the probes TaqLint2 (and respective flanking primers PFLint2 and PRLint2). Mismatches in sequences are highlighted. The GenBank access numbers from which the partial sequences were retrieved are indicated for each species.

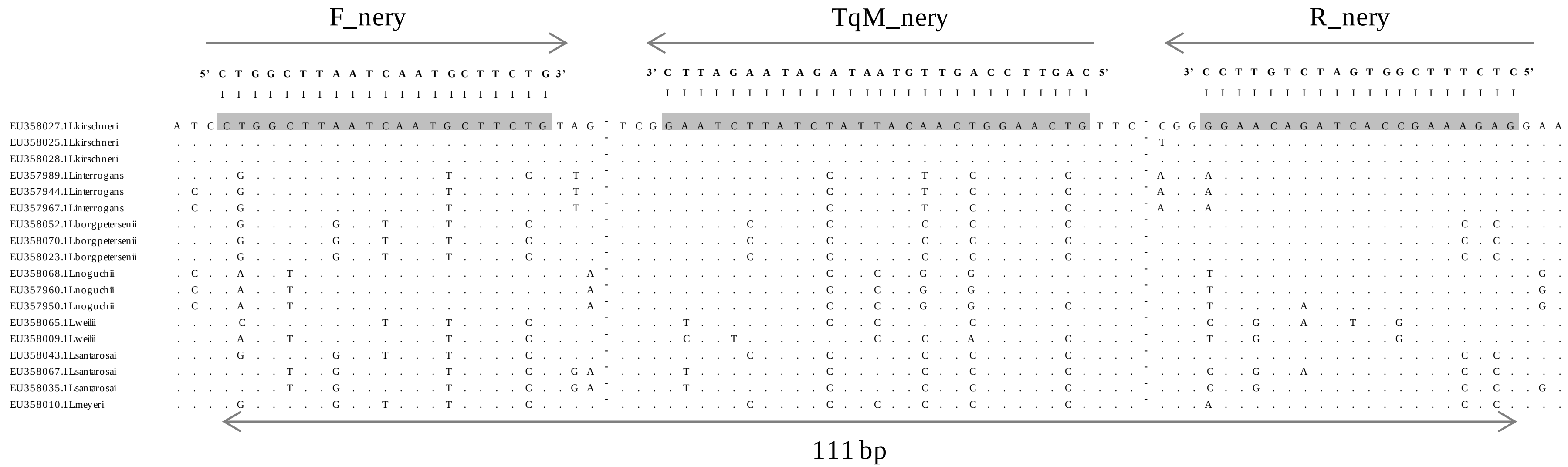

Figure S2. **Complementary targets of the species-specific *Leptospira kirschneri* probe and respective flanking primers.** Partial alignment of *secY* gene sequences of representative *Leptospira* species showing the complementary targets of the probe TqM_nery (and respective flanking primers F_nery and R_nery). Mismatches in sequences are highlighted. The GenBank access numbers from which the partial sequences were retrieved are indicated for each species.

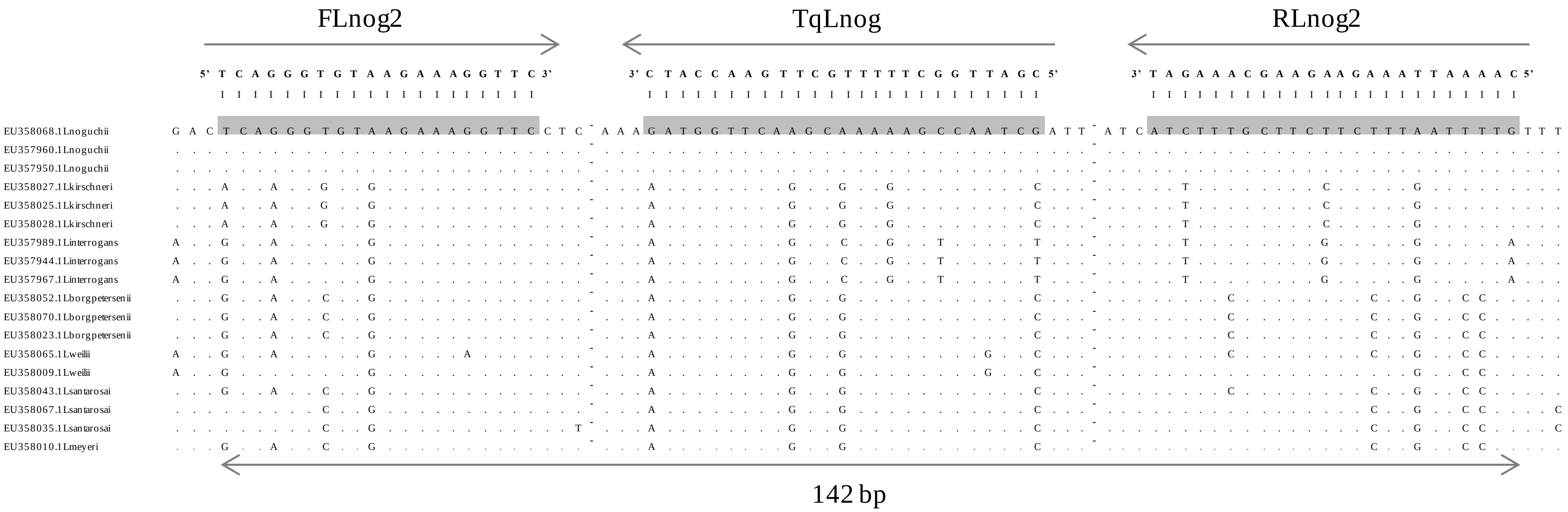

Figure S3. Complementary targets of the species-specific *Leptospira noguchii* probe and respective flanking primers. Partial alignment of *secY* gene sequences of representative *Leptospira* species showing the complementary targets of the probe TaqLnog (and respective flanking primers FLnog2 and RLnog2). Mismatches in sequences are highlighted. The GenBank access numbers from which the partial sequences were retrieved are indicated for each species.

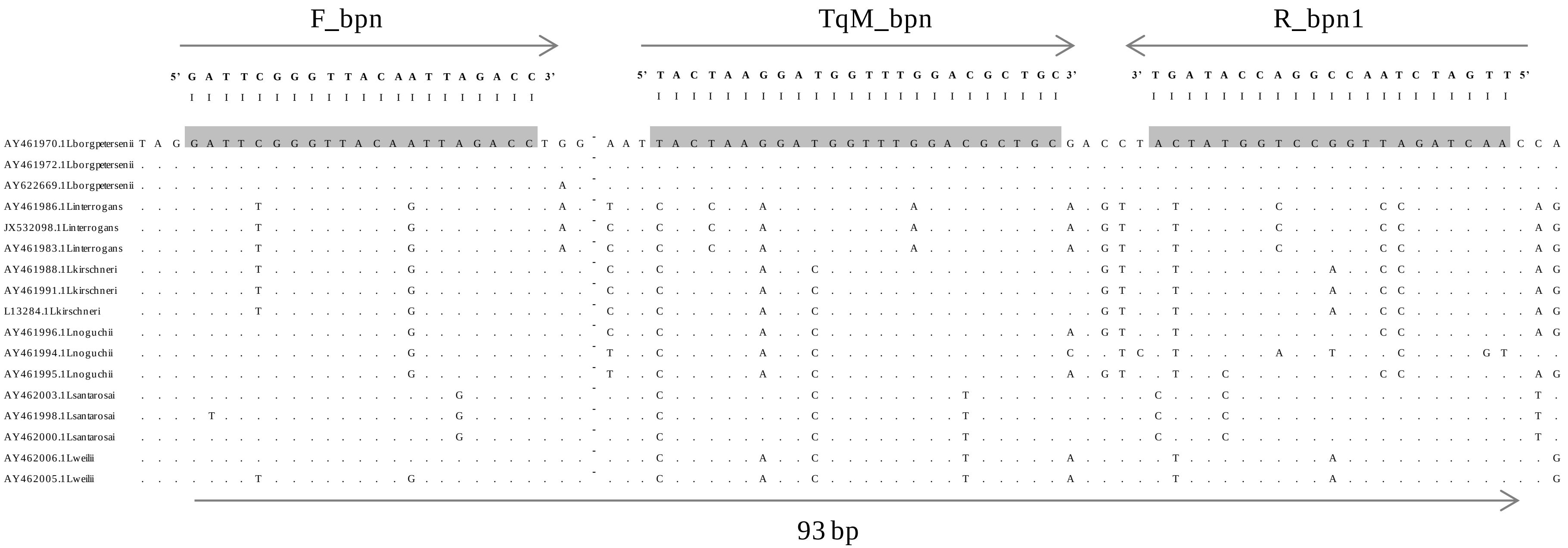

Figure S4. Complementary targets of the species-specific *Leptospira borgpetersenii* probe and respective flanking primers. Partial alignment of *ompL1* gene sequences of representative *Leptospira* species showing the complementary targets of the probe TqM_bpn (and respective flanking primers F_bpn and R_bpn1). Mismatches in sequences are highlighted. The GenBank access numbers from which the partial sequences were retrieved are indicated for each species.
